# A Standardized 10-Step Algorithm for Vitrectomy in Diabetic Tractional Retinal Detachment

**DOI:** 10.7759/cureus.99850

**Published:** 2025-12-22

**Authors:** David Pérez González

**Affiliations:** 1 Medical and Surgical Retina, Centro Latino Israelí de Medicina Ocular, Tulancingo, MEX; 2 Ophthalmology, Clínica Montzerrat, Tulancingo, MEX

**Keywords:** diabetic retinopathy, diabetic tractional retinal detachment, diabetic vitrectomy, pars plana vitrectomy, type 2 diabetes

## Abstract

This review outlines a standardized surgical strategy for managing tractional retinal detachment (TRD) secondary to proliferative diabetic retinopathy. TRD remains a challenging complication due to dense fibrovascular adhesions, persistent posterior hyaloid traction, and a high risk of intraoperative bleeding and iatrogenic retinal breaks. A structured approach is essential to enhance surgical control and reduce complications. The technique emphasizes controlled vitreous removal, early hemostasis, identification of safe dissection planes, careful handling of fibrovascular tissue, and complete elimination of posterior hyaloid remnants. Visualization is optimized with selective adjuncts, followed by comprehensive laser treatment and systematic peripheral inspection to ensure retinal stability. By prioritizing deliberate tissue manipulation and thorough treatment of ischemic retina, this standardized method aims to minimize traction, prevent intraoperative and postoperative complications, and improve anatomical consistency in complex diabetic TRD cases. Although visual recovery remains limited by the underlying disease, applying a consistent and methodical strategy can enhance predictability and overall surgical outcomes.

## Introduction

Tractional retinal detachment (TRD) represents one of the gravest complications of proliferative diabetic retinopathy (PDR). Even in the era of improved glycemic control and widely available treatments, many patients with PDR still progress to TRD, risking severe and often irreversible visual loss [1]. According to recent literature, anatomical reattachment rates after pars plana vitrectomy (PPV) for diabetic TRD are high, with failure rates of around 5.9% after a single surgery; however, despite good reattachment, functional visual outcomes often remain guarded, owing to underlying ischemic and fibrotic damage to the retina [2]. These findings highlight the continuing need for refined surgical techniques, emphasizing minimal retinal manipulation, complete removal of vitreous scaffolding, careful membrane dissection, and comprehensive treatment of ischemic retina.

In light of this, a concise, reproducible 10-step surgical technique for diabetic TRD vitrectomy is described. This technique is designed to minimize iatrogenic traction, maximize membrane removal, ensure retinal reattachment, and reduce postoperative complications.

## Technical report

Surgical technique: 10 concise steps

Core Vitrectomy With Dissection of Mid-periphery Vitreous

Perform a core vitrectomy and deliberately dissect the vitreous in the mid-periphery relative to the posterior pole. This maneuvre helps to limit traction on membranes at the posterior pole. The rationale is that the peripheral vitreous acts like a “basket” attached to the vitreous body - if left intact, manipulation during membrane peeling may transmit traction forces to the retinal periphery and predispose to iatrogenic tears.

Coagulation (endodiathermy) of Active Fibrovascular Membranes

Before manipulating membranes, apply endodiathermy/diathermy coagulation to active fibrovascular membranes. This helps avoid active bleeding during their dissection, improving visualization and reducing the risk of hemorrhagic complications.

Identification of the Dissection Plane

Carefully identify a safe dissection plane between the membranes and the underlying retina or posterior hyaloid. Proper plane identification is critical to avoid inadvertent retinal breaks or mechanical trauma.

Membrane Cutting and Dissection Using High-Speed Vitrector

Use the vitrector to cut and dissect membranes. Increase the cut rate (e.g., ≥ 7000 cuts per minute) and use a vacuum setting no greater than 350 mmHg (or the equivalent in your system), to allow gentle, controlled removal of tissue. As you dissect the posterior hyaloid from the retina, pay special attention to areas with fibrovascular adhesions: preferentially cut those first rather than exert tangential traction, thus reducing risk of retinal tears.

Induction of Posterior Vitreous Detachment or PVD (if Not Already Present)

Once the membranes are separated, you may induce a PVD, if not present. Identify residual vitreoretinal adhesions, particularly in areas of previous fibrovascular attachment, and cut them before proceeding to full detachment - to avoid generating traction forces on the retina.

Completion of Vitrectomy (Clean-Up)

After PVD induction and membrane removal, perform a thorough clean-up. Remove residual cortical vitreous, any vitreous skirt, and ensure adequate vitreous base shaving if indicated (depending on lens status, anterior hyaloid status, and risk factors).

Injection of Triamcinolone to Reveal Residual Posterior Hyaloid or Vitreous Cortex

Instill triamcinolone into the vitreous cavity to highlight any residual posterior hyaloid or cortical vitreous. This improves visualization and ensures completeness of vitreous removal, which is essential to prevent postoperative traction or proliferation. Preservative-free triamcinolone, such as Triesence® (Harrow, Inc., Nashville, US) comes as a 1 mL vial containing 40 mg/mL. Surgeons typically inject 0.1 mL (≈4 mg) into the vitreous cavity for visualization. When using Kenalog® (Bristol Myers Squibb, New Jersey, US) off-label, the preservative is removed by a compounding pharmacy to obtain a non-preserved 40 mg/mL suspension, which is often diluted 1:4 with balanced salt solution (BSS) before use [3].

Pan-Photocoagulation Endolaser

Perform panretinal photocoagulation (PRP) using an endolaser. Given that PDR is the underlying pathology, PRP remains a core goal of the surgery. Adequate and extensive laser helps reduce risk of postoperative neovascularization, recurrent hemorrhage, and further proliferation.

Peripheral Indentation to Check for Iatrogenic Retinal Breaks/Tears

Use scleral indentation to carefully inspect the peripheral retina under wide-view visualization. Confirm there are no iatrogenic retinal breaks, tears, or areas at risk before concluding surgery.

Fluid-Air Exchange, Use Additional Laser if Needed

Perform a fluid-air exchange. During the air fill, the periphery often becomes more visible - if any peripheral areas remain under-treated, apply additional laser photocoagulation as needed, especially in the context of ischemic retina, retinal thinning, or prior proliferative changes.

Please watch Video 1 to revisit these steps and develop a more concise representation of the technique:

**Video 1:** Ten essential steps for performing pars plana vitrectomy (PPV) in proliferative diabetic retinopathy

## Discussion

Vitrectomy for TRD secondary to PDR is widely recognized as one of the most challenging procedures in vitreoretinal surgery [1]. The combination of fibrovascular proliferation, tenacious posterior hyaloid adherence, and ischemic retinal fragility creates an environment in which even well-executed maneuvers can lead to intraoperative bleeding or iatrogenic retinal breaks [1,3]. These factors underscore the need for a structured and deliberate surgical approach that minimizes traction, stabilizes the surgical field, and reduces avoidable complications.

The technique described in this article aligns with the growing emphasis on controlled vitreous removal and progressive membrane dissection advocated in contemporary vitreoretinal surgery [4]. By focusing on the early control of hemostasis, identification of safe dissection planes, and careful segmentation and delamination of fibrovascular tissue, the surgeon can limit the transmission of tractional forces to the retina. This is particularly important given that the subtle traction transmitted through adherent hyaloid or fibrovascular plaques remains a primary cause of intraoperative retinal tears in TRD surgery [3].

Adjunctive measures, such as the use of triamcinolone for visualization and high-cut/low-vacuum vitrectomy settings, have been increasingly adopted to enhance precision and reduce turbulence during tissue manipulation [5]. These strategies contribute to more complete removal of the posterior hyaloid scaffold, addressing a fundamental driver of postoperative proliferation and recurrent traction [1,2,4].

Thorough peripheral evaluation remains another essential pillar of modern TRD surgery. Missed iatrogenic peripheral breaks continue to be a preventable cause of postoperative rhegmatogenous detachment, and wide-field visualization combined with scleral indentation significantly improves detection [3]. Similarly, comprehensive panretinal photocoagulation during the same surgical session provides stability by reducing the ischemic stimulus that drives neovascular proliferation, supporting a more favorable postoperative course [6] .

In summary, the structured surgical approach presented here integrates widely accepted principles of TRD surgery with a logical, reproducible sequence of maneuvers. Although visual outcomes in diabetic TRD remain limited by the underlying severity and chronicity of the disease, maximizing anatomical success and minimizing avoidable complications are crucial in optimizing long-term results. Standardization also facilitates teaching, reproducibility, and further refinement of technique.

## Conclusions

The described 10-step technique offers a structured, reproducible approach to vitrectomy for diabetic tractional retinal detachment. Emphasizing mid to peripheral vitrectomy at the start, cautious membrane dissection, use of high-cut rate vitrectomy, careful induction of PVD, and thorough vitreous cleanup. This method aims to minimize iatrogenic traction and retinal breaks. Complemented with triamcinolone staining, meticulous endolaser PRP, and careful peripheral inspection, the technique seeks to maximize anatomical reattachment and reduce postoperative complications.

Although anatomical success rates (reattachment) after PPV for diabetic TRD are high, functional visual outcomes remain guarded, with many patients ending with relatively poor visual acuity. Therefore, surgical excellence and early referral (before irreversible retinal damage) remain critical determinants of final visual prognosis. 
